# Whey Protein Ingestion Activates mTOR-dependent Signalling after Resistance Exercise in Young Men: A Double-Blinded Randomized Controlled Trial 

**DOI:** 10.3390/nu1020263

**Published:** 2009-12-14

**Authors:** Michelle M. Farnfield, Kate A. Carey, Petra Gran, Marissa K. Trenerry, David Cameron-Smith

**Affiliations:** Molecular Nutrition Unit, Deakin University, Burwood, Victoria, 3125, Australia; Email: Michelle.Farnfield@y7mail.com (M.M.F.); kate.carey@austin.org.au (K.A.C.); petra@deakin.edu.au (P.G.); marissa.trenerry@deakin.edu.au (M.T.)

**Keywords:** leucine, BCAA, p70^S6K^, 4E-BP1, resistance exercise

## Abstract

The effect of resistance exercise with the ingestion of supplementary protein on the activation of the mTOR cascade, in human skeletal muscle has not been fully elucidated. In this study, the impact of a single bout of resistance exercise, immediately followed by a single dose of whey protein isolate (WPI) or placebo supplement, on the activation of mTOR signalling was analyzed. Young untrained men completed a maximal single-legged knee extension exercise bout and were randomized to ingest either WPI supplement (*n* = 7) or the placebo (*n* = 7). Muscle biopsies were taken from the *vastus lateralis* before, and 2, 4 and 24 h post-exercise. WPI or placebo ingestion consumed immediately post-exercise had no impact on the phosphorylation of Akt (Ser^473^). However, WPI significantly enhanced phosphorylation of mTOR (Ser^2448^), 4E-BP1 (Thr^37/46^) and p70^S6K^ (Thr^389^) at 2 h post-exercise. This study demonstrates that a single dose of WPI, when consumed in modest quantities, taken immediately after resistance exercise elicits an acute and transient activation of translation initiation within the exercised skeletal muscle.

## 1. Introduction

Resistance exercise is a potent stimulus of muscle hypertrophy, with net increases in skeletal muscle protein synthesis persisting during recovery [1,2]. Necessary for the enhanced rate of protein synthesis is the activation of kinases in the IGF-1/Akt/mTOR pathway [3,4]. mTOR exerts a critical role in mediating the signaling necessary for mRNA translation initiation, an important regulatory site in cellular protein synthesis [5]. Key targets for mTOR activation include the 70-kDa ribosomal protein S6 kinase (p70^S6K^) and the eukaryotic initiation factor 4E-binding protein (4E-BP1). Both are crucial in stimulating downstream effectors in the activation of the ribosomal subunits and the maintenance of translation for 5’-terminal polypyrimidine (TOP sequences) mRNA’s, that encode many ribosomal proteins and translation factors integral to accommodate demands of increased protein synthesis. 

The activation of translation initiation following acute resistance exercise in the absence of nutrient ingestion is minimal [6,7]. However, both the activation of skeletal muscle protein synthesis and translation initiation is markedly enhanced with the ingestion of essential amino acids. The activation of translation initiation by amino acids is independent of upstream components of IGF-1 signalling, with mTOR acting as a convergence point for the separate actions of amino acids and resistance exercise [5,8]. Thus evidence has accumulated such that when a resistance exercise stimuli is combined with a large ingested dose of amino acids, there is synergistic activation of mTOR and downstream kinases necessary for translation initiation [6,7]. In these studies, branch-chain amino acids (BCAA; total dose 100 g) consumed prior, during and in the early recovery phase, resulted in markedly greater mTOR, p70^S6K^ and rpS6 phosphorylation, than was observed with exercise alone. 

The actions of amino acids to potentiate protein synthesis in the recovery period after exercise also extend to the ingestion of whole protein sources, including the dairy proteins casein and whey [9]. Whey proteins are a common source for supplemental amino acids used by body-builders and strength training athletes, yet few studies have addressed whether the ingestion of a dose of whey protein in quantities normally consumed by strength training athletes is effective in activating the key kinase involved in translation initiation [10,11]. Doses of 20 grams of whey protein ingested either immediately prior to or following a single bout of resistant exercise have been previously reported to enhance the rate of protein synthesis [12]. Thus, the present study aimed to measure the phosphorylation status of Akt, mTOR, 4E-BP1, p70^S6K^ and rpS6 in human skeletal muscle following either a single bout of resistance exercise alone (placebo) or when supplemented with a comparable dose of whey protein isolate (WPI). 

## 2. Results and Discussion

### 2.1. Resistance Exercise

Participant characteristics are shown in Table 1. All subjects performed three sets of 12 repetitions of maximal knee extension on the Cybex machine. The groups were matched for age, BMI and leg strength. 

**Table 1:** Subject characteristics and Peak Torque.

	Placebo	WPI
	(n = 7)	(n = 7)
**Age, yr**	23.0 ± 0.9	21.9 ± 0.8
**Height, cm**	178.0 ± 3.5	176.7 ± 2.7
**Mass, kg**	76.7 ± 4.5	76.2 ± 2.8
**BMI, kg·m^2^**	24.1 ± 0.1	24.5 ± 1.1
**Peak Torque (Nm)**		
**Concentric**	212.3 ± 13.3	213.1 ± 10.9
**Eccentric**	260.7 ± 16.2	255.7 ± 9.9

### 2.2. Amino Acid Analysis of Supplements

As BCAA have been identified as potent promoters of protein synthesis above other amino acids, the BCAA composition of the WPI is reported in Table 2. BCAA comprised 25% of the total amino acids measured and specifically leucine contributed 14% of the total amino acids consumed by the WPI group. No amino acids were detected in the placebo supplement. 

**Table 2:** Amino acid composition of WPI drink.

Amino acid g / 200 mL	WPI drink
*Essential amino acids*	
Histidine	0.56
Isoleucine	1.49
Lysine	2.87
Methionine	0.71
Phenylalanine	0.93
Threonine	1.30
Leucine	3.66
Valine	1.41
*Nonessential amino acids*	
Alanine	1.57
Arginine	0.73
Aspartate	2.94
Glutamate	4.80
Glycine	0.39
Proline	1.30
Serine	1.01
Tyrosine	0.95
**Total Amino Acids (g)**	**26.6**
**Total BCAA**	**6.6**

### 2.3. Kinase Analysis

**Figure 1:**
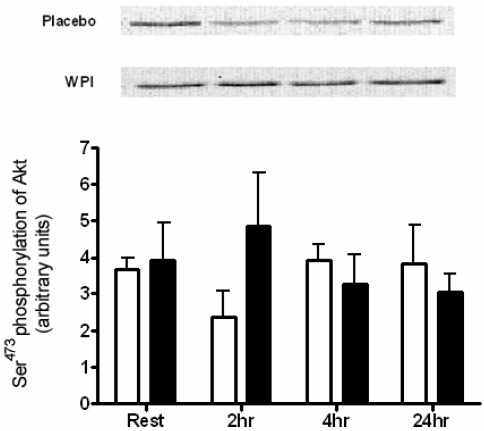
Phosphorylation of Akt at Ser^473^ in human skeletal muscle at rest and 2, 4 and 24 h post-exercise during placebo and WPI trials. Values are arbitrary units (mean ± SEM; n = 7 per trial).Open bars represent placebo, closed bars represent WPI.

A single bout of maximal knee extension exercise, immediately proceeded by either WPI or placebo ingestion had no impact on the phosphorylation of Akt at Ser^473^ (Figure 1). In contrast, 2 h following WPI ingestion, mTOR demonstrated enhanced phosphorylation at the Ser^2448^ site (2.1-fold change).

**Figure 2:**
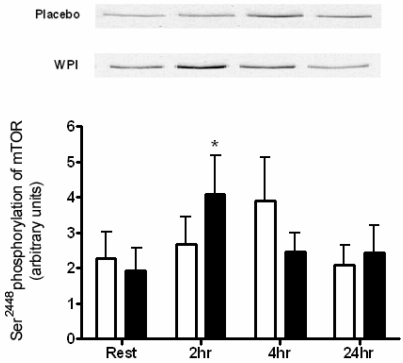
Phosphorylation of mTOR at Ser^2448^ in human skeletal muscle at rest and 2, 4 and 24 h post-exercise during placebo and WPI trials. Values are arbitrary units (mean ± SEM; n=7 per trial). Open bars represent placebo, closed bars represent WPI. *P < 0.05 *vs.* resting values.

**Figure 3:**
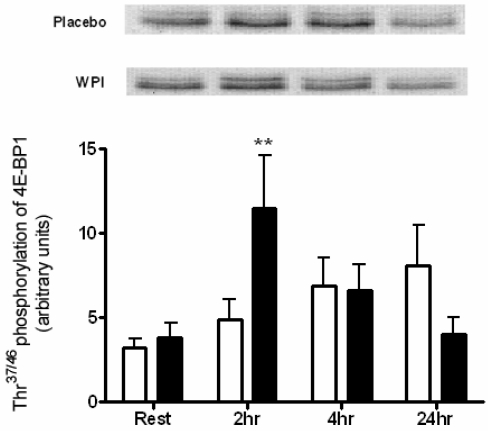
Phosphorylation of 4E-BP1 at Thr^37/46^ in human skeletal muscle at rest and 2, 4 and 24 h post-exercise during placebo and WPI trials. Values are arbitrary units (mean ± SEM; n=7 per trial). Open bars represent placebo, closed bars represent WPI. **P < 0.01 *vs.* resting values.

There was a rise in mTOR Ser^2448^ 4 h following the ingestion of the placebo, though this failed to achieve significance (Figure 2). Of the possible downstream targets of mTOR, the phosphorylation of 4E-BP1 at Thr^37/46^ was significantly increased 3.0-fold above resting values by WPI ingestion (Figure 3). 

**Figure 4:**
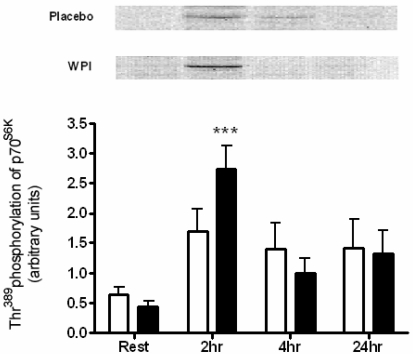
Phosphorylation of p70^S6K^ at Thr^389^ in human skeletal muscle at rest and 2, 4 and 24 h post-exercise during placebo and WPI trials. Values are arbitrary units (mean ± SEM; n = 7 per trial). Open bars represent placebo, closed bars represent WPI. ***P < 0.001 *vs.* resting values.

**Figure 5:**
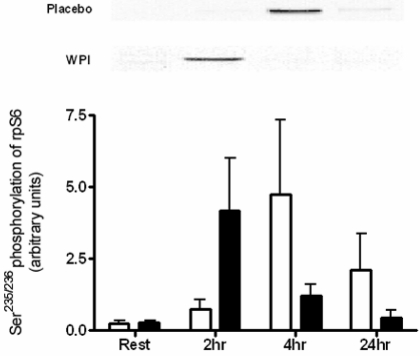
Phosphorylation of rpS6 at Ser^235/236^ in human skeletal muscle at rest and 2, 4 and 24 h post-exercise during placebo and WPI trials. Values are arbitrary units (mean ± SEM; n = 7 per trial). Open bars represent placebo, closed bars represent WPI.

An alternative substrate for mTOR, p70^S6K^ was highly phosphorylated in response to WPI ingestion at 2 h recovery, increasing 6.2-fold (Figure 4). In turn rpS6, the downstream substrate of p70^S6K^, displayed high within-subject variability and failed to achieve significant changes at any time point (Figure 5). 

This study demonstrates the stimulatory effect of WPI supplementation on the activation of regulators of translational initiation, the key step in the commencement of cellular protein synthesis, following a single bout of resistance exercise. Ingestion of WPI (26.6 g) immediately at the completion of a single bout of strenuous leg resistance exercise, induced a greater response in the phosphorylation of mTOR, p70^S6K^ and 4E-BP1, compared to exercise plus placebo ingestion. The heightened responsiveness of these kinases was rapid, predominately occurring within 2 h of exercise completion. Furthermore, there was no evidence for a persistence of this beneficial action for WPI, at both 4 and 24 h following the exercise. 

Resistance exercise provokes muscle hypertrophy through a number of purported signal transduction pathways, including activation of the PI(3)K/Akt pathway [13]. This is evident in both rat and *in vitro* studies [3,14,15]. Akt is a serine/threonine kinase involved in the regulation of cellular metabolism and has been shown to induce rapid skeletal muscle hypertrophy *in vivo* [16]. Phosphorylation of Ser^473^ is required for maximal activation of Akt and it appears that Akt may have a relatively short activation period after an acute bout of resistance exercise [17,18]. Human studies investigating the activation of Akt after resistance exercise are scarce and have shown an enhanced phosphorylation of Akt 1 h post-exercise, but not immediately after exercise [19,20]. Indeed, the supplementary ingestion of 100 g BCAA and exercise combined has been reported to induce a small, albeit significant, increase in Akt activation at 1 hr post-exercise [7]. In the current study, Ser^473^ phosphorylation of Akt was unaltered following either exercise or exercise combined with 26.6 g WPI ingestion at any of the measured time points. 

mTOR is a 289 kDa serine/threonine kinase partially downstream of Akt and responsible for the complex integration of anabolic stimuli mediating cell growth. Chiefly, mTOR stimulates protein synthesis through downstream activation of p70^S6K^ and 4E-BP1, providing a key point of convergence for both resistance exercise and amino acids [5,8]. WPI ingestion significantly increased Ser^2448 ^phosphorylation of mTOR 2 h post-exercise. Interestingly, the ingestion of 100 g BCAA as previously reported, prompted an increase in mTOR phosphorylation at 1 h post-exercise, but failed to persist to 2 h recovery [7]. Both leucine and Akt activate mTOR through phosphorylation of the Ser^2448^ site [8,21]. Amino acids have been described as “priming” molecules, whose phosphorylation of mTOR at Ser^2448^ is a prerequisite for further phosphorylation by Akt [22]. Furthermore, activation of mTOR can occur through two separate signalling pathways by either insulin induced activation of the PI(3)K/Akt pathway or an undefined Akt-independent pathway stimulated by amino acids [23,24,25]. One possible candidate is Vps34 (vacuolar protein sorting mutant 34), a class III PI3K (phosphoinositide 3-kinase), regulated by both resistance exercise and nutrient availability [26]. However the interaction between supplementary protein ingestion and resistance exercise on the regulation of Vps34 is not yet described.

Downstream of mTOR, activation of p70^S6K^ is strongly linked to muscle hypertrophy [27]. p70^S6K^ undergoes phosphorylation at multiple serine and threonine residues, with the Thr^389^ residue closely associated with complete activation of the kinase [28]. The acute resistance exercise bout performed by the placebo group was not sufficient to induce phosphorylation of p70^S6K^ during the time points measured. The provision of whey proteins in this study stimulated an early and robust activation of p70^S6K^. These findings are consistent with those reported previously, in which BCAA ingestion increases phosphorylation of p70^S6K^ at Thr^389^ 2 h post-exercise [6]. Further scrutiny of these results revealed that the magnitude of response was far greater (approximately double) in this earlier trial. This may be due to the large 100 g dose of BCAA ingested, compared to 26.6 g of whey proteins administered in the current study. A dose-response of increasing leucine ingestion to increasing p70^S6K^ (Thr^389^) phosphorylation was observed in rats [29]. Moreover, it is suggested that full activation of p70^S6K^ is dependent on the provision of amino acids to elicit a downstream activation of rpS6 [6,30]. Thus, a modest dose of 26.6 g WPI taken in conjunction with a single bout of resistance exercise clearly changed the signalling response of p70^S6K^, although larger doses of WPI may be required to have a downstream impact on rpS6 during exercise recovery. Exercise plus placebo also resulted in an increased, but not significant, phosphorylation of rpS6, however this occurred later in recovery at 4 h. It is unknown whether this time differential in rpS6 activation is of physiological relevance. 

In addition to p70^S6K^, the phosphorylation of 4E-BP1 is sensitive to amino acid provision [31]. The site-specific phosphorylation of Thr^37/46^ is reported to be crucial for optimal activation of 4E-BP1 [32]. In this study a marked increase in 4E-BP1 phosphorylation at 2 h post-exercise was measured when subjects were fed WPI. No change in Thr^37/46^ phosphorylation of 4E-BP1 was shown after exercise alone. This is consistent with a previous study that not only demonstrated no increase in 4E-BP1 phosphorylation in human skeletal muscle after an acute resistance bout, but a significant decrease in Thr^37/46^ phosphorylation was observed immediately and 2 h post-exercise [30]. 

Whey proteins are a rich source of leucine (14%) and BCAA (26%) [33]. Previously our group has demonstrates that this modest dose (26.6g) WPI elicits a rapid rise in plasma amino acids, peaking around 1 hour following ingestion [34]. Consistent with this data and the demonstration that kinases central to translation initiation are enhance, data has been reported that the ingestion of supplementary WPI, either before or immediately following resistance exercise training enhances both muscularity and strength gains in young males undertaking resistance training programs [35,36,37]. In supporting these studies, our data provides cellular evidence of the efficacy of combined WPI ingestion and resistance exercise to activate translation initiation. Indeed, when consumed immediately at exercise cessation, a modest 26.6 g WPI induced a more rapid and heightened activation of three kinases, mTOR, 4E-BP1 and p70^S6K^ at 2 h recovery. This has important implications for athletes, as this dose can be readily incorporated into a variety of either beverage (as used in the current study) or food formulations. Future research must then be directed towards examining the chronic actions on either muscular mass or strength with equivalent doses of whey protein. 

## 3. Experimental Section

### 3.1. Subjects

Fourteen healthy young men (aged 18–25 yr) who had not participated in regular resistance exercise within a year prior to commencing the study and were not currently using nutritional or other purported muscle building supplements were recruited. A medical history questionnaire was used to exclude subjects with a diagnosed condition or illness that would endanger subjects during strenuous exercise. All subjects were informed of the nature and possible risks of the experimental procedures, before their written informed consent was obtained. This study and all associated procedures was formally approved by the Deakin University Human Research Ethics Committee. 

### 3.2. Nutritional Composition of Supplement Drinks

Subjects were randomly assigned to receive either a whey protein isolate (WPI) or placebo drink. The WPI powder contained 97% protein powder (of which 90% protein, 0.5% fat, 0.5% lactose, 5% moisture, 3.7% ash and 1.15% milk minerals), 2.9% artificial flavour and 0.1% aspartame sweetener, with a dose of 28.65 g powder prepared in 200 mL water (this dose delivered 26.6g protein per 200 mL, see Table 2). The protein powder component was a β-lactoglobulin-enriched whey protein isolate (WPI: NatraBoost^®^ BLG, Murray Goulburn Nutritionals, Brunswick, Victoria, Australia). The placebo powder was prepared in the same volume and contained the same quantity of artificial flavour and aspartame sweetener and both drinks looked identical. 

### 3.3. Amino Acid Analysis of Supplement Drinks

The WPI and placebo supplements were analysed for amino acid composition as described previously [38]. Briefly, samples were hydrolysed in 6 M HCl to determine total amino acids (excluding tryptophan, cysteine, asparagine and glutamine). The hydrolysates were rotary evaporated to dryness to remove the HCl and reconstituted in sodium citrate buffer (pH 2.2), including internal standard norleucine. Samples were then run on HPLC (Water Inc.; Empower Pro, Version 2, Software, Waters Corporation) and amino acids were separated by ion-exchange column (Sodium, Part# WAT08002, Waters, USA) and detected by UV after a post-column ninhydrin reaction. 

### 3.4. Experimental Design

At least four days prior to the testing day, each subject completed a familiarization session on the Cybex NORM dynamometer (Cybex International Inc. UK). For the 24 h preceding, and the days of the trial, subjects consumed a standard controlled diet of 20% fat, 14% protein and 66% carbohydrate and abstained from alcohol, caffeine, tobacco and recreational exercise. Following an overnight fast, subjects reported to the exercise physiology laboratory for a resting muscle biopsy. Thirty minutes after the biopsy subjects completed three sets of 12 repetitions of maximal singled-legged knee extension exercise on the Cybex machine with a 2 minute rest between each set. Each repetition involved concentric and eccentric contraction of the knee extensors at a set speed of 60°/sec on the biopsied leg. Subjects were instructed to contract as hard as possible and were verbally encouraged throughout each set. On completion of the exercise, subjects immediately ingested the supplement and were required to remain resting in the laboratory for biopsies to be taken 2 h and 4 h post-exercise. Subjects were supplied with lunch and an evening meal to eliminate bias from their post-exercise meal and given instructions to avoid caffeine, dairy products and high protein foods (including eggs, chicken, fish and red meat). Subjects then returned to the laboratory the following morning following an overnight fast for a biopsy 24 h post-exercise. Needle biopsies were performed in the *vastus lateralis* muscle of the non-dominant leg, under aseptic conditions following local anesthesia (1% xylocaine). Excised muscle tissue from each biopsy was immediately frozen and stored in liquid nitrogen for subsequent analysis. To minimize the potential or interference of repeated biopsies, samples were collected at least 2 cm from previous biopsy sites. 

### 3.5. Tissue Processing

Muscle samples (~30 mg) were homogenized in ice cold lysis buffer containing 20 mM Tris-HCL, 5 mM EDTA, 10 mM Na-pyrophosphate, 100 mM NaF, 2 mM Na_3_VO_4_, 1% igepal, 10 μg/mL aprotinin, 10 μg/mL leupeptin, 3 mM benzamidine and 1 mM PMSF. Homogenates were rotated on ice for 1 h and then centrifuged at 13,000 x g for 10 min. The resulting supernatant was analyzed for total protein content using the BCA protein assay (Pierce Biotechnology, Inc. Rockford, IL). 

### 3.6. Immunoblot Analysis

Denatured total proteins from each sample in loading buffer were separated by electrophoresis by 5% (phospho-mTOR), 8% (phospho-Akt, phospho-p70^S6K^) or 12% (phospho-4E-BP1, phospho-S6) SDS-PAGE and transferred to nitrocellulose membranes, or PVDF for phospho-4E-BP1 and phospho-S6. PVDF membranes were blocked by air drying and nitrocellulose membranes were blocked with 5% bovine-serum albumin in Tris-buffered saline for 1 h (phospho-mTOR) or 2 h (phospho-Akt, phospho-p70^S6K^). All membranes were incubated overnight at 4°C with polyclonal rabbit anti-phospho-mTOR (Ser^2448^), phospho-p70^S6K^ (Thr^389^), phospho-S6 ribosomal protein (Ser^235/236^), phospho-4E-PB1 (Thr^37/46^) and phospho-Akt (Ser^473^) antibodies at 1:1,000 dilution (all from Cell Signaling Technology, Danvers, MA). Membranes were washed in TBST followed by 60 min incubation with anti-rabbit IgG conjugated to horse-radish peroxidase (Santa Cruz Biotechnology, Santa Cruz, CA) then washed again. Immunoreactive bands were detected using enhanced chemiluminescence (Western Lightning Chemiluminescent Reagent, Perkin-Elmer, Boston, MA) and visualized on a Kodak Image Station 440CF, using Kodak ID 3.5 image analysis software (Perkin Elmer Life Sciences, Boston, MA).

### 3.7. Statistical Analysis

Statistical analysis was performed using GraphPad Prism 4.1 (GraphPad Software, San Diego, CA). Means were compared using two-way ANOVA (repeated measures) and any significant differences analyzed using a Bonferroni post hoc test. Data is presented as mean ± standard error of the mean (SEM). A probability level of <0.05 was adopted throughout to determine statistical significance unless otherwise stated.

## 4. Conclusions

The present study provides evidence that the ingestion of a modest dose of whey protein, taken immediately after resistance exercise, is able to enhance the earlier activation of translational kinases in young males compared with exercise alone. Further investigations are required to delineate the timing, formulation and optimal dosage of WPI to induce maximal translation initiation activation. 

## Supplementary Files

Supplementary File 1: Correction (PDF, 17 KB)
