# Correction: Farnfield, M.M., *et al*. Whey Protein Ingestion Activates mTOR-dependent Signalling after Resistance Exercise in Young Men: A Double-Blinded Randomized Controlled Trial. *Nutrients* 2009, *1*, 263-275. 

**DOI:** 10.3390/nu2030317

**Published:** 2010-03-08

**Authors:** Michelle M. Farnfield, Kate A. Carey, Petra Gran, Marissa K. Trenerry, David Cameron-Smith

**Affiliations:** Molecular Nutrition Unit, Deakin University, Burwood, Victoria, 3125, Australia; Email: Michelle.Farnfield@y7mail.com (M.M.F.); kate.carey@austin.org.au (K.A.C.); petra@deakin.edu.au (P.G.); marissa.trenerry@deakin.edu.au (M.T.)

We found an error in our paper recently published in *Nutrients* [1]. In our discussion of the Karlsson *et al.* 2004 paper published in the *American Journal of Physiology* [2], we failed to identify that the dose of branched-chain amino acids (BCAA) was calculated on the basis of body weight. These investigators provided the subjects a total of 100 mg BCAA/kg body weight, not a total ingested dose of 100 g as stated in our paper. 

This error impacts on the conclusions we draw, as our paper refers to this dose as ‘large’, when in actual fact, the dose of BCAA administered in the Karlsson *et al.* 2004 paper (calculated, approx. 7.44g) [2], is similar to the 6.6g BCAA amount provided in our study, delivered in 26.6g whey protein isolate [1]. 

We apologise for any inconvenience caused to the readers.
