# Supplementary material for: Whey Protein Ingestion Activates mTOR-dependent Signalling after Resistance Exercise in Young Men: A Double-Blinded Randomized Controlled Trial
Source: Nutrients. 2009 Dec 14;1(2):263–75. doi: 10.3390/nu1020263 (PMC3257597; doi:10.3390/nu1020263)
Supplement: Supplementary File 1: — Correction (PDF, 17 KB) [file nutrients-01-00263-s001.pdf]

Nutrients **2010**, *2*, 317-318; doi:10.3390/nu2030317

OPEN ACCESS

nutrients

ISSN 2072-6643

www.mdpi.com/journal/nutrients

Correction

Correction: Farnfield, M.M., *et al*. Whey Protein Ingestion Activates mTOR-dependent Signalling after Resistance Exercise in Young Men: A Double-Blinded Randomized Controlled Trial. *Nutrients* 2009, *1*, 263-275.

Michelle M. Farnfield, Kate A. Carey, Petra Gran, Marissa K. Trenerry and David Cameron-Smith *

Molecular Nutrition Unit, Deakin University, Burwood, Victoria, 3125, Australia;
E-Mails: Michelle.Farnfield@y7mail.com (M.M.F.); kate.carey@austin.org.au (K.A.C.); petra@deakin.edu.au (P.G.); marissa.trenerry@deakin.edu.au (M.T.)

* Author to whom correspondence should be addressed: E-Mail: davidcs@deakin.edu.au; Tel.: +61-3-9244 6502; Fax: +61-3-9244 6017.

Received: 8 March 2010 / Published: 8 March 2010

We found an error in our paper recently published in *Nutrients* [1]. In our discussion of the Karlsson *et al.* 2004 paper published in the *American Journal of Physiology* [2], we failed to identify that the dose of branched-chain amino acids (BCAA) was calculated on the basis of body weight. These investigators provided the subjects a total of 100 mg BCAA/kg body weight, not a total ingested dose of 100 g as stated in our paper.

This error impacts on the conclusions we draw, as our paper refers to this dose as 'large', when in actual fact, the dose of BCAA administered in the Karlsson *et al.* 2004 paper (calculated, approx. 7.44g) [2], is similar to the 6.6g BCAA amount provided in our study, delivered in 26.6g whey protein isolate [1].

We apologise for any inconvenience caused to the readers.

References

1. Farnfield, M.M.; Carey, K.A.; Gran, P.; Trenerry, M.K.; Cameron-Smith, D. Whey protein ingestion activates mTOR-dependent signalling after resistance exercise in young men: A double-blinded randomized controlled trial. *Nutrients*. **2009**, *1*, 263-275.

2. Karlsson, H.K.R.; Nilsson, P.A.; Nilsson, J.; Chibalin, A.V.; Zierath, J.R.; Blomstrand, E. Branched-chain amino acids increase $p70^{S6k}$ phosphorylation in human skeletal muscle after resistance exercise. *Am. J. Physiol. Endocrinol. Metab.* **2004**, *287*, 1-7.

© 2010 by the authors; licensee Molecular Diversity Preservation International, Basel, Switzerland. This article is an open-access article distributed under the terms and conditions of the Creative Commons Attribution license (http://creativecommons.org/licenses/by/3.0/).
